# Neuroendocrine-Immune Crosstalk Shapes Sex-Specific Brain Development

**DOI:** 10.1210/endocr/bqaa055

**Published:** 2020-04-09

**Authors:** Sheryl E Arambula, Margaret M McCarthy

**Affiliations:** 1 Department of Pharmacology, University of Maryland School of Medicine, Baltimore, MD; 2 Program in Neuroscience, University of Maryland School of Medicine, Baltimore, MD

**Keywords:** sex differences, neuroimmune, neurodevelopment, hormone, microglia, cytokines

## Abstract

Sex is an essential biological variable that significantly impacts multiple aspects of neural functioning in both the healthy and diseased brain. Sex differences in brain structure and function are organized early in development during the critical period of sexual differentiation. While decades of research establish gonadal hormones as the primary modulators of this process, new research has revealed a critical, and perhaps underappreciated, role of the neuroimmune system in sex-specific brain development. The immune and endocrine systems are tightly intertwined and share processes and effector molecules that influence the nervous system. Thus, a natural question is whether endocrine-immune crosstalk contributes to sexual differentiation of the brain. In this mini-review, we first provide a conceptual framework by classifying the major categories of neural sex differences and review the concept of sexual differentiation of the brain, a process occurring early in development and largely controlled by steroid hormones. Next, we describe developmental sex differences in the neuroimmune system, which may represent targets or mediators of the sexual differentiation process. We then discuss the overwhelming evidence in support of crosstalk between the neuroendocrine and immune systems and highlight recent examples that shape sex differences in the brain. Finally, we review how early life events can perturb sex-specific neurodevelopment via aberrant immune activation.

Crosstalk between the neuroendocrine and immune systems during the critical period of sexual differentiation is an important mechanism by which sex differences in the brain are established. Bidirectional communication between these 2 physiological systems occurs molecularly through a common biochemical language composed of membrane-derived signaling molecules, neurotransmitters, cytokines, hormones, and their respective receptors. Many of these shared signaling molecules shape sexually differentiated endpoints in the brain, including neurogenesis, synaptic formation and pruning, and programmed cell death. Although the immune system is emerging as a critical regulator of brain development, we have only scratched the surface of understanding neuroendocrine-immune communication during sex-specific brain development, and much remains unknown. In this mini-review, we first operationally define categories of sex differences in the brain and discuss specific examples that illustrate their distinction. Next, we provide an overview of sexual differentiation of the brain, which occurs during the perinatal period, and the powerful influence of steroid hormones on this process. We then focus on identified sex differences in the developing neuroimmune system and review the overwhelming evidence for neuroendocrine-immune crosstalk, known mechanisms of communication, and highlight recent examples of neuroendocrine-immune crosstalk that influence sex differences in the brain. Finally, we discuss how early life events can influence sex-specific brain development via aberrant immune activation.

## Types of Sex Differences in the Brain

**Figure 1.**
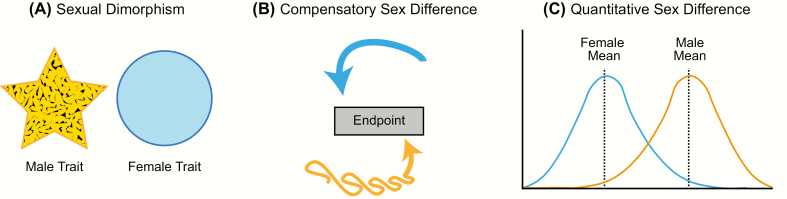
Types of sex differences. **A:** Sexual dimorphism is when a particular trait (neuroanatomical, physiological, or behavioral) has 2 distinct forms in males and females that do not overlap in form or function (yellow and blue shapes representing each sex differ in form). **B:** Compensatory sex difference is when males and females display the same behavior or physiology, but the processes underlying this trait differ in each sex (depicted by yellow and blue arrows). **C:** Sex difference is when a trait exists on the same continuum in males and females, but the average differs between the sexes.

The influence of sex on brain structure and function is pervasive and, contrary to long-held assumptions, expands beyond the neural control of reproductive behaviors into every aspect of the healthy and diseased brain. Psychiatric and clinical literature reinforces the importance of sex differences in the brain, as numerous neurodevelopmental disorders exhibit strong sex biases in incidence, severity, and progression. However, before considering the origins of sex differences in the brain, it is necessary to first classify types of sex differences (Fig. 1). The categories defined below are not exclusive but rather provide the necessary framework to evaluate and interpret the relative impact of a particular sex difference (1, 2).

The first type, sexual dimorphism, is the appropriate term for cases in which the male and female trait (morphological, physiological, or behavioral) differs markedly in form. The word “dimorphism” derives from the Latin word “dimorphos,” which means “occurring in two different forms.” In scientific literature, however, this term is frequently used as a general catch-all to describe sex-related brain differences. Unfortunately, this imprecise and inconsistent use generates considerable confusion and controversy; consequently, studies on sex differences are often oversimplified and taken out of context, resulting in headlines such as “Sex: The Real Reason Men Are Better at Reading Maps” (3). In reality, both sexes are equally skilled at spatial cognition tasks, but they employ different strategies in order to achieve the same result (4–7). True neural and behavioral dimorphisms are exceptionally rare in humans, but a few cases associated with sex-typical reproductive behaviors have been identified in other species. One example is the song system of songbirds, a discrete network of brain nuclei necessary for song learning and production. Male songbirds sing intricate songs to attract potential mates and defend their territory from intruders, and male song is more complex and frequent than female song in the majority of songbird species (8). Although there is variation between species in the degree of sexual dimorphism, several nuclei in the male song system are markedly diminished or even missing in females (9). For instance, in zebra finches, where only males sing, all nuclei in the song system are substantially larger in volume in males than females, and the striatal song nuclei known as Area X is absent in females (10–12).

The second type of sex difference, termed “compensation” or “convergence,” is perhaps the most overlooked because it involves instances in which the 2 sexes converge to the same endpoint from different beginnings. Simply put, a compensatory sex difference is when males and females display the same behavior or physiology, but the processes underlying this trait differ in each sex. Thus, sex differences in the brain may promote or restrict functional differences between males and females (13). One widely cited example is the marked sex difference in vasopressin innervation of the forebrain. Adult males have significantly more vasopressin neurons within the bed nucleus of the stria terminalis and the medial amygdala, and a higher density of vasopressin innervation projecting from these regions to the forebrain than females (14). Evidence from the biparental prairie voles suggests these sex differences promote care-giving behavior in males, given that they do not experience pregnancy or parturition, and therefore reduce sex differences in the amount of parental care provided to the offspring (15). Perinatal steroid hormone exposure establishes the number of vasopressin neurons and vasopressin innervation in the brains of males (16, 17). More recently, research on the molecular mechanisms of memory formation has revealed numerous sex differences in signaling pathways and transcription factors that are recruited in the hippocampus during memory consolidation (18–22). While our current understanding of sex differences in the molecular mechanisms of memory is limited, males and females perform equally well across a wide variety of cognitive tasks despite sex differences in how these tasks are processed. Converging evidence suggests that differential recruitment of signal transduction pathways serves to make the sexes more alike, resulting in broadly similar memory performance among males and females (23). Examples of compensatory sex differences have also been reported in the immune system, where the male-sex chromosome complement (ie, XY) appears to counteract the immunosuppressive effects of circulating testosterone in adult males (24, 25).

The final and most common type of sex differences are quantitative and continuous, meaning the distribution for a particular endpoint may overlap substantially between males and females, but the mean consistently differs. Social behavior, stress and anxiety responses, pain sensitivity, and drug addiction are all well-established examples of behavioral traits that differ, on average, between males and females but show varying degrees of overlap. In this review, we will use the term “sex difference” to refer to any neuroanatomical, physiological, or behavioral trait that is different in males and females but varies along a continuum.

## Origins of Sex Differences in the Brain

Sex differences in the brain are widespread and found at every level of biological complexity, from noncoding small RNAs that regulate gene expression to the ultimate output of the nervous system: behavior. Some sex differences in the brain are macroscopic and quite profound, as in the case of entire brain regions that vary in size according to sex (26). But the overwhelming majority are far more subtle, yet no less important. For instance, the signal transduction pathways mediating memory formation differ between males and females (23). Sex differences in the brain are integral to almost every aspect of neural functioning and, therefore, the importance of studying their origins cannot be overstated. Moreover, biological sex is a major determinant of the prevalence and severity of many neuropsychiatric disorders, response to brain injury, and predilection for drug addiction (2, 27, 28).

The embryonic brain begins as a bipotential organ, equally capable of taking on a male-typical or female-typical phenotype. Decades of research led to the prevailing dogma that sexual differentiation of the brain occurs in a stepwise fashion and depends on gonadal steroids. Simply stated, if the fetus has inherited a Y chromosome, testes will develop and secrete gonadal steroid hormones that act directly on the brain to produce male-typical neuroanatomy. Female-typical neuroanatomy, in contrast, develops in the relative absence of gonadal steroid hormones (29). While recent findings on local steroidogenesis in the brain challenge the relative simplicity of this gonad-centric dogma, steroid hormones are indeed the major source of sex differences in the brain (26, 30–32). Regardless of where they are produced, steroid hormones permanently influence the neural structure in a sex-specific manner during a restricted developmental window. The actions of steroid hormones on the brain during this time are referred to as “organizational.” Sexually differentiated, or organized, neural substrates are then activated in adulthood by steroid hormones to produce male- or female-typical functions and behaviors (33). In rodents, estrogens locally aromatized from fetal testicular androgens are essential for many—but not all—aspects of brain masculinization (34, 35). Gonadal hormones also contribute to human neural and behavioral sex differences, but evidence from nonhuman primates and humans indicates that androgens, not estrogens, appear to drive masculinization in primates (36).

## Sex Differences in the Developing Neuroimmune System

### Innate immune cells of the brain

Microglia are best known for being the brain’s first line of defense against infection or injury, monitoring the central nervous system (CNS) parenchyma for danger, clearing debris, and mediating inflammation and repair with the production of prostaglandins, nitric oxide, reactive oxygen species, and cytokines, among other diffusible messengers (37–42). Beyond their duties as CNS sentinels, they also play critical roles in the development and function of the nervous system. Microglia facilitate the establishment of neural circuits by participating in neurite growth, promoting cell genesis, controlling cell numbers, synaptic pruning, and apoptosis (38, 43–45).

In contrast to other CNS cells, which arise from the neuroectoderm, microglia originate from yolk-sac macrophage precursors that migrate to the brain during early embryonic development, where they take up permanent residence (46–48). In the developing brain, microglia are dynamic and can rapidly change their function and morphology in response to their local microenvironment (49). “Ramified” microglia have long, delicate processes with which they survey the brain parenchyma and “ameboid-like” microglia have enlarged cell bodies with shortened and fewer processes. Although the “ameboid-like” morphology denotes a greater production of inflammatory signaling molecules and the “ramified” morphology denotes surveillance of neighboring cells, microglia function/activational state is not exclusive to either form (50). Sex differences in microglia morphology and number manifest after the perinatal testicular androgen surge, and they vary by brain region, suggesting an organizational role of hormones (51). Sex differences in microglia number and/or morphology exist in multiple regions of the neonate brain, including the amygdala, medial preoptic area (POA), and hippocampus (43, 51–53). These morphological sex differences may reflect sex differences in functional or maturational state.

Sex differences in microglia function during development and organize the neural circuitry controlling sexually differentiated behaviors. Juvenile social play is a rewarding behavior performed by most mammalian species, including humans, that is important for social, emotional, and cognitive development. Social play is most common during adolescence but males play more vigorously and frequently than females. In the neonatal medial amygdala of males, the perinatal testosterone surge enhances endocannabinoid tone, inducing microglia to engulf and eliminate newborn astrocytes. Females, in contrast, have a lower endocannabinoid tone and subsequently, less microglial phagocytosis occurs, allowing more astrocytes to survive to the juvenile age. Blocking phagocytosis by microglia in the medial amygdala of males results in an increased number of astrocytes and a corresponding reduction in social play (53). Microglia phagocytosis has also been found to mediate the natural decrease in social play at the end of adolescence by eliminating dopamine receptors in the nucleus accumbens of male rats (54).

Mast cells are another class of innate immune cells that are best known for their ability to mediate inflammation by releasing histamine and other substances into the surrounding tissue during a process called degranulation (55). They originate from hematopoietic stem cells in the bone marrow and become dispersed throughout the entire body, where they are strategically positioned as gatekeepers within tissues interfacing with the external environment (56–58). Mast cells are not restricted to the periphery, however, and brain-resident populations are typically found around the meninges, third ventricle, the choroid plexus, and the parenchyma of the thalamus and hypothalamus (59). Brain-resident mast cells produce numerous mediators implicated in brain development, including serotonin and other neurotransmitters, histamine, cytokines, and growth factors, and can communicate with microglia (59–61). In the POA of neonates, mast cells are more numerous and more active in males than females (62). Recent evidence indicates that mast cells may be underappreciated regulators of sex-specific neurodevelopment and will be reviewed further below (see Neuroendocrine and Immune System Communication).

### Cytokines

Cytokines are a category of small signaling molecules that act as messengers among immune cells to mediate numerous functions. Initially discovered in the immune and hematopoietic systems, cytokines were once thought to be produced solely by monocytes and lymphocytes, but it is now evident they are produced by virtually every nucleated cell in the body (63). In the brain, cytokines are best known for initiating and propagating immune responses in various pathological conditions. However, as our understanding of cytokines has evolved, it is now clear they also play critical neuromodulatory roles unrelated to trauma or infection (64).

The healthy brain expresses a variety of cytokines and their receptors, and nearly all CNS cells produce and respond to cytokines (eg, neurons, microglia, oligodendrocytes, and astrocytes) (65–67). Cytokine receptors have been identified throughout the developing brain; however, relative densities for individual receptors are dynamic and vary by brain region and age (68, 69). While the homeostatic role of most cytokines remain unstudied in the CNS, recent research demonstrates that several cytokines are critical for various aspects of neurodevelopment, including neurogenesis, migration, proliferation, differentiation, and synaptic maturation and pruning. Because the role of cytokines in normal brain development has been reviewed extensively elsewhere (64, 70), we limit our discussion to those involved in sex-specific brain development for brevity. The 6 major types of cytokines are interleukins (IL), chemokines, colony-stimulating factors, tumor necrosis factors (TNF), interferons, and transforming growth factors. Traditionally, cytokines are classified as either pro- or anti-inflammatory, depending on whether they promote or suppress inflammation; however, this classification is not absolute, as the local environment, synergistic or competing factors, receptor density, and tissue responsiveness also determine the net inflammatory response to cytokines (71).

The IL-1 superfamily consists of 11 cytokines and 10 receptors that play a central role in initiating and regulating innate and adaptive inflammatory responses to a wide range of stimuli (72, 73). The most extensively studied among these are the potent proinflammatory cytokines IL-1α and IL-1β, which were initially recognized for their pyrogenic properties, and the IL-1 receptor agonist (IL-1Ra) that inhibits IL-1α and IL-1β activity by competitively binding to the IL-1 type 1 receptor (74). Outside of the context of injury and infection, emerging evidence indicates that locally-produced IL-1 members are key regulators of brain development and homeostasis and participate in major neurodevelopmental events, including neurogenesis, astrocyte lineage commitment, and the modulation of microglia activity (75–79). Throughout brain development, several members of the IL-1 family exhibit sex-biased expression patterns that appear to be highly dependent on age and brain region. One particularly exhaustive study characterized the gene expression of various immune molecules important for microglia recruitment and function in the hippocampus and parietal cortex of male and female rats at 3 developmental timepoints (birth, neonatal, and adult) (51). In the hippocampus and the parietal cortex, expression of *Il-1Ra* and *Il-36Ra*, both anti-inflammatory receptor agonists that inhibit IL-1 signaling, are significantly higher in females across all 3 representative timepoints. Sex differences in the proinflammatory cytokines IL-18 and IL-1α are age-dependent, with adult males having higher expression of *Il-18* in both regions and adult females having higher expression of *Il-1a* in both regions. Microglia cultures derived from male and female neonatal mouse brains also exhibit basal sex differences in cytokine expression, as cultures from females have higher expression of IL-6, a member of the IL-6 cytokine family, and IL-1β than cultures from males (80).

Chemokines, a subtype of cytokines with chemoattractant properties, are of central importance to neurodevelopment because they direct the proliferation and migration of immature neurons, glia, and microglia and modulate axon pathfinding (81, 82). Marked sex differences in chemokine subfamilies CC and CXC exist during early postnatal development in the amygdala, hippocampus, and parietal cortex (51). For example, *Ccl20* and *Ccl14* expression is significantly upregulated in the hippocampus and parietal cortex of males at birth when compared to females, but this sex difference is no longer present by 4 days of age. At this same age, expression of *Cxcl9* is undetectable in the hippocampus but females have nearly 30-fold higher expression of *Cxcl9* than males in the amygdala.

Given the very few number of studies examining basal sex differences in cytokine expression in the developing brain, along with our nascent understanding of nonclassical actions of cytokines, it remains unclear how these sex differences may affect CNS physiology during brain development or later in adulthood. Moreover, further research is needed to dissect the hormonal contribution to sex differences in immune signaling molecules.

## Neuroendocrine and Immune System Communication

One of the first studies suggesting neuroendocrine-immune crosstalk dates to the 1930s, when Hans Selye observed adrenal hypertrophy and thymus involution in stressed animals (83), an effect later attributed to the immunosuppressive actions of adrenal glucocorticoids (84). Despite this, the neuroendocrine and immune systems remained independent fields of study, each with its own jargon and few researchers fluent in both. It was not until the late 1970s that truly interdisciplinary research, exploring the interactions between behavioral, neural, endocrine, and immune processes gained momentum (85, 86). In the time since then, extensive research has irrefutably established that the neuroendocrine and immune systems are physiologically connected and interact via dynamic, bidirectional pathways. The immune system is not autonomous, it acts in concert with the neuroendocrine system to maintain homeostasis by sensing and counteracting changes in the brain and periphery throughout the lifespan of an organism. Anatomical and molecular mechanisms underlying these bidirectional neuroendocrine-immune interactions are summarized comprehensively elsewhere (86–89); therefore, we only briefly outline basic principles of this crosstalk.

**Figure 2.**
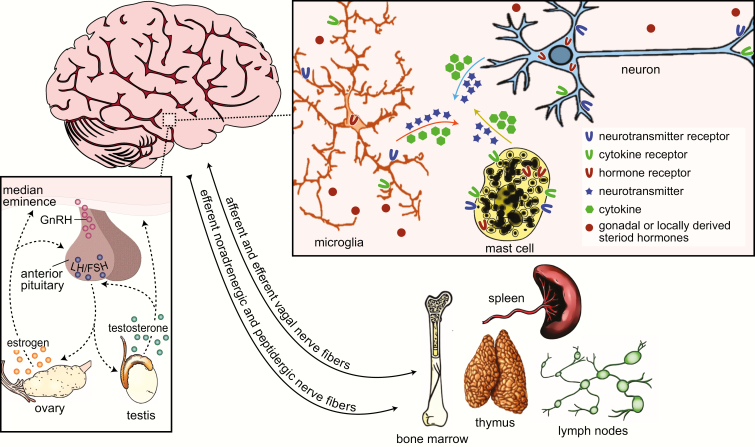
Bidirectional communication between the neuroendocrine and immune systems. Crosstalk between the neuroendocrine and immune systems is enabled by shared signaling molecules that include neurotransmitters, hormones, cytokines, and their respective receptors, among others (top-right inset). Neurotransmitters and hormones act by binding to and activating receptors on target cells of the endocrine and nervous systems and brain-resident and peripheral immune cells. Likewise, cytokines and their receptors are expressed by many cells in the central nervous system and they modulate immune responses locally and in the periphery. Primary and secondary lymphoid organs of the immune system, such as the bone marrow, spleen, thymus, and lymph nodes are innervated by noradrenergic and peptidergic nerve fibers, and to some extent vagal nerve fibers (bottom right). Remaining relatively quiescent until adolescent, the hypothalamic-pituitary-gonadal axis (HPG; bottom-left inset) is the primary regulator of the mature reproductive system. Environmental cues trigger the release of gonadotropin-releasing hormone (GnRH) from neurons in the preoptic area and hypothalamus to the median eminence. GnRH causes the release of luteinizing hormone (LH) and follicular-stimulating hormone (FSH) from the anterior pituitary, which, in turn, stimulates the secretion of steroid hormones from the gonads. These steroid hormones then feedback to the anterior pituitary and steroid-sensitive hypothalamic neurons to regulate HPG activity.

While physically separated, the neuroendocrine and immune systems are connected by a common biochemical language, nerve fibers, and the lymphatic system (Fig. 2). Communication between and within the neuroendocrine and immune systems is enabled by a shared repertoire of bioregulatory signals that includes neurotransmitters, hormones, cytokines, and their respective receptors (90–92). Immune cells can both respond to and secrete neurotransmitters and hormones, while CNS and endocrine cells produce cytokines that can modulate immune responses locally and in the peripheral nervous system (87, 93–95). Both primary and secondary lymphoid organs of the immune system, such as the bone marrow, thymus, lymph nodes, and spleen, are innervated by noradrenergic and peptidergic nerve fibers (96). Bidirectional communication is further facilitated by the recently discovered meningeal lymphatic system, which drains cerebrospinal and interstitial fluids from the brain parenchyma into the deep cervical lymph nodes and allows the circulation of immune cells from the CNS and meninges to the periphery (97).

**Figure 3.**
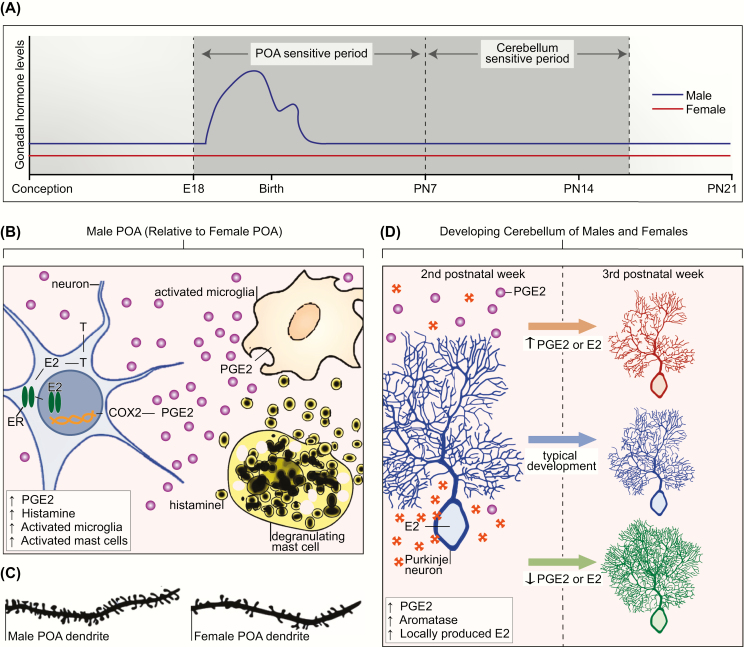
**A:** Sensitive periods for differentiation of the preoptic area of the hypothalamus (POA) and cerebellum in the laboratory rat. Sensitive periods are related to critical periods in that they are times when development can be permanently derailed by exogenous or endogenous stimuli. The sensitive period for sexual differentiation of the POA is defined in the rat by the onset of androgen production by the fetal male testis on embryonic day 18 (E18) and the loss of sensitivity to exogenous steroid hormones in females by the end of the first postnatal week (postnatal day 7 [PN7]). In the cerebellum, Purkinje neurons show dramatic growth and differentiation during the first 3 postnatal weeks of life. There is a sensitive period during the second postnatal week of life during which disruption of estradiol (E2) or prostaglandin (PGE2) dysregulates cerebellar Purkinje neuron development. Changes to PGE2 or E2 levels in either the first or third postnatal week has no impact on Purkinje neuron growth. **B:** Crosstalk between the neuroendocrine and immune systems establish sex differences in the POA. In the developing male POA, E2 aromatized from testicular androgens (T) binds to estrogen receptor alpha (ER) and induces transcription of the cyclooxygenase enzyme (COX2), the rate-limiting enzymes in PGE2 synthesis. PGE2 triggers microglia to produce still more PGE2. Mast cells further facilitate PGE2 synthesis by degranulating and releasing histamine, which activates the neighboring microglia. Glutamate release from astrocytes (not depicted), stimulated by PGE2, is an essential last step in this series of coordinated events to organize the formation of dendritic spine synapses on POA neurons, which are 2-fold higher in males than females (**C**). **D:** Cerebellum development is mediated by neuroendocrine to immune communication. Generally, the cerebellum is not considered a sexually differentiated brain region. In both sexes, endogenous PGE2 is elevated during the second postnatal week, and this drives a parallel increase in local E2 production by stimulating activity of the aromatase enzyme. During this period, deviation in either direction of the levels of PGE2 or E2 has deleterious consequences for Purkinje neurons. If production is increased, Purkinje neuron growth is stunted. If production is decreased, Purkinje neurons show excessive growth of the dendritic tree. Both responses have long-term consequences for behavior.

As described previously, sex differences in the developing neuroimmune system are evident in the number and activation states of microglia and mast cells and the expression of chemokines and cytokines. To illustrate neuroendocrine-immune communication that contributes to normal brain development, we will focus on 2 regions: the POA and the cerebellum (Fig. 3). They are both developmentally regulated by the inflammatory signaling molecule prostaglandin E2 (PGE2) but in different ways. The POA exhibits sex differences in a variety of morphological and physiological parameters that are central the expression of adult male sexual behavior and female maternal behavior. To recap, during late embryonic development the fetal testis produces high levels of androgens that gain access to the brain, where they are converted to estrogens and initiate brain masculinization; thus, the male POA is exposed to higher levels of androgens and estrogens than the female POA during the perinatal sensitive period. In the male POA, estradiol induces the expression of cyclooxygenase-2 (COX2), the major enzyme responsible for prostanoid synthesis in the brain, which preferentially promotes the synthesis of PGE2 (98, 99). PGE2 activates a signal transduction cascade that results in the formation and stabilization of dendritic spine synapses on POA neurons (100–102). Subsequently, the resulting density of spine synapses per unit of dendrite is twice as great in males when compared to females, and this permanent organizational sex difference is positively correlated with the expression of adult male copulatory behavior (103). In the neonatal POA, microglia are more numerous and, on average, exhibit a more ameboid-like or activated state in males than females. Microglia are the major source of PGE2 and in the male POA they amplify PGE2 signaling by making still more PGE2 in a positive feed-forward mechanism. Mast cells also facilitate PGE2 synthesis by microglia (43). In the male, POA mast cells are more numerous than in females, and estradiol causes mast cells to degranulate and release histamine, which then triggers microglia to produce PGE2 (62). The PGE2-mediated induction of male-typical synaptic patterning in the POA and the resulting male-typical copulatory behavior are prevented by pharmacologically disrupting the PGE2 feed-forward signaling during the first postnatal week of life (43, 62, 99, 104).

In contrast to the POA, the cerebellum is generally not considered a sexually differentiated brain region; however, it provides an example of a naturally occurring neurodevelopmental trajectory that is modulated by immune-to-neuroendocrine communication. Cerebellar PGE2 levels are endogenously high during the second postnatal week in the rodent, and this stimulates aromatase activity in Purkinje neurons, leading to a parallel increase in locally produced estradiol. Disruption of either PGE2 synthesis or estradiol during this time has detrimental consequences on cerebellar Purkinje neuron dendritic arborization. In both sexes, treatment with PGE2 or estradiol during the second postnatal week stunts Purkinje neuron dendritic length and complexity. Blocking PGE2 or estradiol production during this time has the opposite effect on Purkinje neurons, resulting in an increase in dendritic length and complexity (105, 106).

These and other studies reveal the potential for crosstalk between the neuroendocrine and neuroimmune mechanisms of neurodevelopment. They also highlight the challenge of unraveling the role of the immune system in brain sexual differentiation, because the signal transduction pathways of specific immune cells and local hormone levels often vary by brain region and age. Although much attention has been given to how endocrine-immune crosstalk mediates sex differences in the POA, there is little knowledge of how this crosstalk influences other brain regions that show sex differences in morphology, physiology, or mediate behavioral sex differences. How and to what degree the complex interplay between the endocrine and immune system regulates normal sex-specific brain development are important areas for future exploration.

## Points of Vulnerability: Reprogramming of Sex-Specific Neurodevelopment by Immune Disruption

Critical periods of development are restricted intervals of time during which some enduring feature of the brain is established, such as a neuroanatomical or a neurophysiological endpoint. Critical periods are characterized by rapid growth and, as a result, are susceptible to disruption by intrinsic and extrinsic factors; even minor deviations from the typical developmental trajectory can have long-lasting or permanent consequences. The critical period for sexual differentiation occurs during early neurodevelopment when the brain is particularly sensitive to steroid hormone exposure (107). In rats, this critical period is well defined and spans the perinatal period, occurring from approximately embryonic day 18 through the first week of postnatal life. Convergent evidence indicates the critical period for sexual differentiation is entirely prenatal in humans (36, 108). The immune system is emerging as a fundamental mediator of sexual differentiation, and subsequently, abnormal immune system activation during this critical period, irrespective of its origin, may impact brain development. In the following sections we discuss environmental factors that aberrantly influence the developing neuroimmune system in a sex-dependent manner.

### Prenatal immune challenge

In rodent models of maternal immune activation (MIA), the 2 most commonly used immunostimulants are the bacterial mimetic lipopolysaccharide (LPS) and the viral mimetic polyriboinosinic polyribocytidylic acid (Poly I:C). Common findings include increased levels of proinflammatory cytokines in the maternal and fetal circulation, as well as in the fetal brain, and subsequent behavioral abnormalities are observed in the offspring (109–112). Sex differences are apparent in the hippocampus as a consequence of prenatal LPS exposure. In mice, the juvenile male offspring of LPS-treated dams have an increased number of dendritic spines on granule cells of the dentate gyrus, and this is paralleled by a reduction in fractalkine receptor expression. In contrast to males, there are no significant effects of prenatal LPS exposure on the spine density of granule cells or the expression of fractalkine receptor in the hippocampus of juvenile female offspring (113). In the CNS, fractalkine is a chemokine constitutively expressed by neurons, and the fractalkine receptor is predominantly found on microglia. Fractalkine signaling mediates communication between microglia and neurons and is crucial for microglia recruitment and microglia-mediated synaptic pruning, suggesting the effects of LPS on hippocampal spine density in males could arise from alterations in microglial function (44, 114). Additional studies, which did not examine females, provide further evidence for deficits in hippocampal microglia following a prenatal immune challenge. Prenatal exposure to Poly I:C increased adult microglial expression of TNF-α and IL-1β in the hippocampus of males. This was correlated with a reduction in adult hippocampal neurogenesis and impairments in sensorimotor gating behavior, which could be reversed by treatment with the microglial inhibitor minocycline (115, 116).

Epidemiological studies and animal models of MIA have substantiated a role for early aberrant immune activation in the risk of developmental neuropsychiatric disorders, with evidence being most robust for schizophrenia and growing for autism spectrum disorders (ASDs) (117, 118). Clinical data indicates that schizophrenia and ASDs are more frequently diagnosed or have more severe clinical features in males than females (119–122). However, the mechanistic basis for this sex-bias remains unknown. Despite the clear utility of the MIA model for understanding the origins of developmental neuropsychiatric disorders, the potential influence of sex has long been ignored (123). Thus, future research is needed in this area to fully interpret and classify the relationship between prenatal immune challenges and sex-specific outcomes in the brain.

### Environmental chemicals

Endocrine-disrupting chemicals (EDCs) are a diverse group of exogenous compounds that interfere with the endocrine system and produce adverse health effects in exposed individuals or their offspring (124). Many classes of chemicals, including plasticizers, flame-retardants, fungicides, pesticides, pharmaceuticals, heavy metals, and even naturally occurring compounds such as phytoestrogens, are EDCs (125). Early life EDC exposure can impair neurodevelopmental outcomes, but few studies to date have investigated the effects of EDCs on immune-related endpoints in the developing brain (126, 127). Prenatal exposure to bisphenol A (BPA), a ubiquitous EDC initially developed as a synthetic estrogen, alters the sex- and region-specific colonization of microglia in 2 rodent models, the rat and the prairie vole (128). Prenatal BPA exposure nearly doubled the total number of microglia found in the dentate gyrus of preweanling female rats (129). Gestational exposure to lead and pesticides also alters microglial numbers and cytokine expression; however, the potential for sex differences in the response has not been explored (130, 131). Fine-particulate air pollution is another environmental exposure that can disrupt the developing neuroimmune system and alter the trajectory of brain development in a sex-specific manner. Acute, prenatal exposure to diesel exhaust particles (DEP), the major component of motor vehicle–related air pollution, increases the production of inflammatory cytokine IL-1β and alters the morphology of microglia in the embryonic rodent brain, but this occurs only in males. Males also exhibit persistent changes in parietal cortex volume in response to prenatal DEP exposure, but the directionality of these changes shift during maturation from an increased volume at embryonic day 18 to a decreased volume at postnatal day 30. The authors attribute this male-specific temporal shift in parietal cortex volume to an impact of DEP on the developing neural stem cell niche. In partial support of this conclusion, males prenatally exposed to DEP have significantly increased numbers of microglia-neuron interactions in the parietal cortex as adolescents, relative to all other groups, but no other timepoints were examined (132). Immune disruption is an emerging area in developmental neurotoxicology, and while there is some evidence of altered neuroimmune responses following developmental EDC exposure, further research is necessary to understand how exogenous chemicals may stimulate an immune response in the developing brain.

### Developmental programming

Epidemiological studies provide compelling evidence of an association between immune system dysfunction and neuropsychiatric disorders that have a distinct etiology in neurodevelopment. Strikingly, many of these disorders display a pronounced sex bias in their prevalence and age of onset, as well as symptom presentation and treatment response. Males are much more likely to have disorders that emerge early in life, whereas females are more likely to develop disorders that arise around the onset of puberty or later. At present, the mechanistic bases for these sex-related differences remain elusive. We hypothesize that males may be more vulnerable because many of the pathways classically associated with inflammation are required for masculinization of the brain. Thus, the heightened inflammatory milieu naturally present in the male brain during the critical period of sexual differentiation puts males at greater risk when aberrant immune activation occurs during early life (133). Overall, there appear to be multiple pathways in which the developing nervous system and the endocrine and immune systems may bias one sex towards disease susceptibility.

## Conclusions

Sexual differentiation of the brain occurs during a narrow developmental window and induces enduring neuroanatomical and physiological changes that profoundly impact neural functioning. Sex differences in brain and behavior are largely dependent on differential exposure to gonadal steroid hormones, but there is strong evidence that the immune system also plays a role in this process. Research on the contribution of the immune system to the development of neural sex differences is still in its early days and many unanswered questions remain. However, it is apparent that the contribution of the immune system to neural sex differences cannot be understood in isolation from the endocrine system. As reviewed above, the immune system interacts with the neuroendocrine system in a bidirectional manner, and this crosstalk has recently emerged as a critical regulator of sex differences in the brain. As our knowledge of neuroendocrine-immune crosstalk and sexual differentiation rapidly advances, it is necessary to recognize that each of these physiological systems can be affected by aberrant or exogenous stimuli, resulting in lasting changes to the brain. Studies aimed at identifying the natural processes by which sex-specific brain development is established provide us an opportunity to define disease mechanisms and identify novel targets for therapy and prevention.

## Additional Information

***Disclosure Summary:*** The authors have nothing to disclose.

***Data Availability.*** Data sharing is not applicable to this article as no datasets were generated or analyzed during the current study.
